# Mesoporous Aerogel Microparticles Injected into the Abdominal Cavity of Mice Accumulate in Parathymic Lymph Nodes

**DOI:** 10.3390/ijms22189756

**Published:** 2021-09-09

**Authors:** Gábor Király, John Chinonso Egu, Zoltán Hargitai, Ilona Kovács, István Fábián, József Kalmár, Gábor Szemán-Nagy

**Affiliations:** 1 Department of Molecular Biotechnology and Microbiology, University of Debrecen, Egyetem tér 1, H-4032 Debrecen, Hungary; gkiralydeb@gmail.com (G.K.); szeman-nagy.gabor@science.unideb.hu (G.S.-N.); 2 MTA-DE ELKH Homogeneous Catalysis and Reaction Mechanisms Research Group, Department of Inorganic and Analytical Chemistry, University of Debrecen, Egyetem tér 1, H-4032 Debrecen, Hungary; chinonsojohnegu@science.unideb.hu (J.C.E.); ifabian@science.unideb.hu (I.F.); 3 Department of Pathology, Kenézy University Hospital, University of Debrecen, 2-28 Bartók Béla Street, H-4031 Debrecen, Hungary; dr.hargitai.zoltan@kenezy.unideb.hu (Z.H.); dr.kovacs.ilona@kenezy.unideb.hu (I.K.)

**Keywords:** aerogel, biocompatibility, biodistribution, toxicity, fluorescein

## Abstract

Mesoporous aerogel microparticles are promising drug delivery systems. However, their in vivo biodistribution pathways and health effects are unknown. Suspensions of fluorescein-labeled silica–gelatin hybrid aerogel microparticles were injected into the peritoneum (abdominal cavity) of healthy mice in concentrations of 52 and 104 mg kg^−1^ in a 3-week-long acute toxicity experiment. No physiological dysfunctions were detected, and all mice were healthy. An autopsy revealed that the aerogel microparticles were not present at the site of injection in the abdominal cavity at the end of the experiment. The histological study of the liver, spleen, kidneys, thymus and lymphatic tissues showed no signs of toxicity. The localization of the aerogel microparticles in the organs was studied by fluorescence microscopy. Aerogel microparticles were not detected in any of the abdominal organs, but they were clearly visible in the cortical part of the parathymic lymph nodes, where they accumulated. The accumulation of aerogel microparticles in parathymic lymph nodes in combination with their absence in the reticuloendothelial system organs, such as the liver or spleen, suggests that the microparticles entered the lymphatic circulation. This biodistribution pathway could be exploited to design passive targeting drug delivery systems for flooding metastatic pathways of abdominal cancers that spread via the lymphatic circulation.

## 1. Introduction

Nowadays, numerous studies have focused on the biomedical applications of aerogels [1]. Aerogels are a group of solid materials of high porosity (90–99%), low density (0.005–0.50 g cm^−3^) and high specific surface area (300–1000 m^2^ g^−1^). They have a highly interconnected open pore structure with adjustable pore size and morphology. Inorganic oxide and biopolymer aerogels are promising candidates for developing novel drug delivery systems (DDS) [2,3]. Even low water solubility drugs can be loaded up to 40 wt.% into aerogels, and these formulations can be utilized in oral, transdermal and nasal administration routes to increase the bioavailability of the low solubility drugs [2,3,4]. Several biopolymer and hybrid aerogels have been investigated as drug delivery systems [5,6,7,8]. Modifications of the structure or the composition of these aerogels offer the possibility of achieving tunable and stimuli-responsive drug release. Aerogels can easily be formulated into microparticles, 10–100 µm in diameter [9,10,11,12,13]. Aerogel microparticles can be useful for delivering covalently conjugated drugs when their suspension is administered by intraperitoneal (i.p.) injection. For example, microparticles injected into the abdominal cavity could possibly follow abdominal tumor cells that metastasize to the parathymic lymph nodes via the lymphatic circulation, providing passively targeted drug delivery [12,14].

The biocompatibility and biodistribution of all new aerogels intended to be used as drug delivery systems have to be investigated in order to provide information on their health effects [15]. In addition, understanding the biodistribution of i.p. injected aerogel particles may facilitate the development of passively targeted delivery systems. Unfortunately, there are only a handful of studies reporting the in vitro biocompatibility of aerogels tested on different cell cultures [13,16,17,18,19,20]. The number of in vivo studies is even lower. These mainly focus on the oral administration of drug-loaded aerogels, and aerogels for tissue regeneration [6,21,22,23,24,25].

Microparticles from silica–gelatin hybrid aerogels have been synthesized to develop passively targeted delivery systems for anti-cancer drugs. Both pristine and fluorescein-labeled silica–gelatin aerogel microparticles (FSGM) were found to be biocompatible in vitro on cell cultures [12,13]. In these cultures, SCC and HaCaT cells flocked into the direction of the hybrid particles in a chemotactic response due to the presence of gelatin. In general, gelatin is a biomaterial that induces cell proliferation, relieves oxidative stress and strengthens the immune system by increasing the thymus index [26,27]. Furthermore, the covalent conjugation of the anticancer drug methotrexate (MTX) into these silica–gelatin hybrid aerogel microparticles results in an anti-cancer effect on tumorous cell cultures [12]. Despite the promising in vitro results, experimental data on the in vivo biocompatibility and biodistribution of silica–gelatin aerogel microparticles are not available. In the case of in vivo investigations, depending on the route of administration, the main questions are the distributive pathways to the target tissues, biocompatibility with healthy tissues and cellular responses at the localized site of the particles.

In this paper, we report details on the biocompatibility and biodistribution pathways of fluorescein-labeled silica–gelatin aerogel microparticles (FSGM) in healthy mice following the i.p. administration of microparticle suspension into the abdominal cavity. The liver, the spleen, the kidneys and the thymus of the mice were prepared and studied combining histological evaluation with immunohistochemistry, and fluorescence microscopy with image analysis. The pathological evaluation of organs is presented to give a complete picture of the health effects of the injected silica–gelatin aerogel microparticles.

The preparation, characterization and controlled release properties of methotrexate (MTX)-containing silica–gelatin aerogel microparticles are detailed in our previous publication [12]. The present study is a standalone investigation of the biocompatibility and biodistribution of the “empty” silica–gelatin aerogel microparticles as potential drug delivery devices. In this aspect, the present study serves as the basis for understanding the passive targeting properties of the hybrid aerogel microparticles as delivery vehicles.

## 2. Materials and Methods

### 2.1. Chemicals

Tetramethyl orthosilicate (TMOS), methanol, acetone and ammonium carbonate ((NH_4_)_2_CO_3_) were purchased from Fluka. Food-grade gelatin (Type A, 150 kDa) was purchased from Dr. Oetker (Bielefeld, Germany). Fluorescein 5(6)-isothiocyanate (FITC) and (3-aminopropyl)trimethoxysilane (APTMS) were purchased from Sigma Aldrich (Budapest, Hungary). Other chemicals (NaCl, HCl, NaOH, NaH_2_PO_4_, Na_2_HPO_4_) were ACS reagent grade (Sigma-Aldrich). All aqueous solutions were prepared with Milli-Q water (Millipore, Budapest, Hungary). Supercritical CO_2_ was produced from min. 99.5% pure gas (Linde, Budapest, Hungary).

### 2.2. Preparation and Characterization of Fluorescein Functionalized Silica–Gelatin Aerogel

Silica–gelatin hybrid aerogel of 20 wt.% gelatin content is synthesized by using the sol–gel technique combined with co-gelation. The silica precursor TMOS is hydrolyzed in an aqueous solution in the presence of dissolved gelatin. The detailed recipe for the pristine silica–gelatin hybrid aerogel of 20 wt.% gelatin content is given in a previous publication [28]. The detailed recipe of the preparation and the complete characterization of the fluorescein functionalized silica–gelatin aerogel is given in another previous publication [13].

Briefly, 10 mg FITC and 16 µL (APTMS) were dissolved in 7.0 mL methanol and stirred at room temperature for 3 h, in order to conjugate fluorescein to APTMS. Next, 3.0 mL TMOS was added to this reaction mixture. Another aqueous solution was prepared from 20.0 g water, 0.60 g gelatin and 70 mg (NH_4_)_2_CO_3_. These two solutions were mixed under intense stirring and poured into a Teflon mold for gelation. The unreacted FITC from the first solution spontaneously reacts with the gelatin content of the mixture. The alcogel was aged for 24 h in the mold, which was followed by a multi-step solvent exchange in order to replace the original solvent mixture with pure acetone. Finally, acetone was extracted from the gel with supercritical CO_2_ at 14 MPa and 80 °C in a pumpless drying system, as described in a technical paper [29].

**Figure 1:**
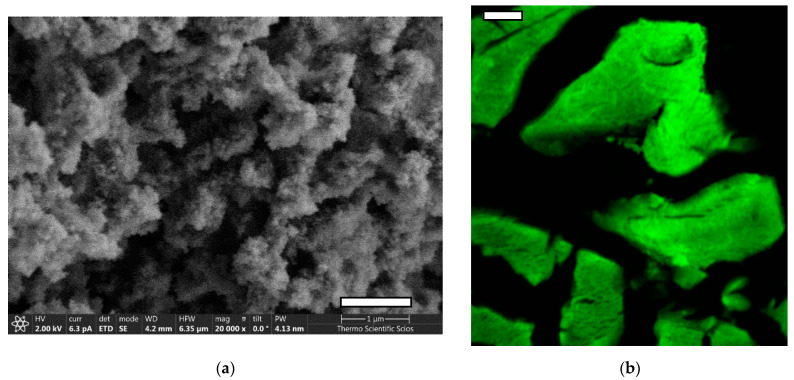
(**a**) Representative SEM image of the fresh fracture surface of fluorescein functionalized silica–gelatin aerogel (FSG). Scale bar: 1 µm. (**b**) Confocal fluorescence microscopy images of paraffin-embedded aerogel particles. Scale bar: 15 µm.

The resulting fluorescein functionalized silica–gelatin aerogel (FSG) has a gelatin content of 20 wt.% by the dry weight. The specific surface area of the aerogel is 402 m^2^/g and the mean pore size is 20 nm according to N_2_-sorption porosimetry analysis (Supplementary: Figure S1). The aerogel is dominantly mesoporous with few macropores. The fluorescence properties of the functionalized aerogel are practically the same as those of fluorescein, as described in our previous publications [12,13,28]. Representative SEM images and confocal fluorescence microscopy images of paraffin-embedded aerogel particles are shown in Figure 1.

### 2.3. Sterilization and Micronization of the Aerogel

First, the functionalized silica–gelatin aerogel (FSG) monoliths were gently ground and sieved (≤100 µm) in their dry state. Then, the aerogel particles were sterilized by UV light. The aerogel particles were placed into a UV transparent plastic container and spread to a maximum thickness of 1–2 mm. The aerogel particles were irradiated with UV light for 24 h in ca. 360°. This sterilization method was successfully used in previous in vitro experiments [12,13].

**Figure 2:**
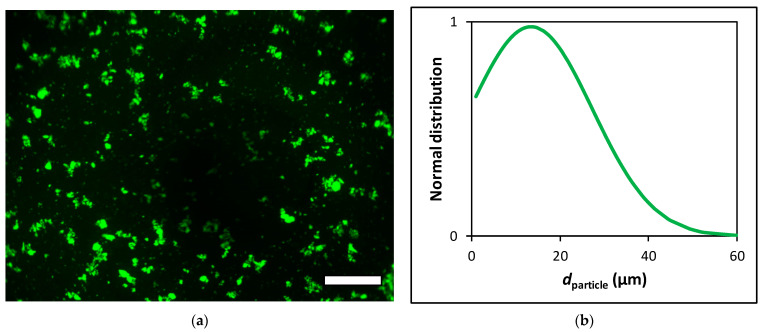
(**a**) Fluorescence microscopy images of the FSGM suspension recorded using FITC fluorescence filter set. Scale bar: 100 µm. (**b**) Size distribution of suspended FSGM determined by image analysis.

After sterilization, the aerogel particles were hydrated by 0.9% saline and micronized using a Potter–Elvehjem tissue grinder. After 10 min of wet grinding, the suspension of the aerogel particles was sonicated in a glass container in a sonicator bath for 10 min. These micronized aerogel particles are termed fluorescein functionalized silica–gelatin aerogel microparticles (FSGM). The particle concentration and size distribution of suspended FSGM were measured using a hemocytometer and digital image analysis. The aqueous FSGM suspension was injected into a Buerker hemocytometer chamber, and digital microscopic images were recorded using 40× dry objectives in an Olympus AX70 fluorescence microscope. Images were collected from 3 evenly distributed focal planes from the 100 µm depth of the counting region. Digital images were analyzed by the National Institute of Health ImageJ software using threshold function for image segmentation. Particle size distribution was quantified using the “Analyze Particles” function on these binary images. Fluorescence microscopy images and particle size distribution curves are shown in Figure 2. For all further experiments, the suspensions of these aerogel microparticles were used in 1.0 or 2.0 mg mL^−1^ concentrations, as calculated from their dry weight.

No aggregates were detected by microscopy in the FSGM suspensions in 4 h after preparation. No fluorescein leaching is detectable from FSGM by spectrofluorimetry over 4 weeks of soaking in aqueous buffers in the pH range from 4.0 to 7.5 [13].

### 2.4. Animals

The C3H mice are widely used in cancer, infectious disease and cardiovascular research areas [30,31]. Herein, 15–22 weeks old male and female C3H mice were kept in a conventional laboratory environment, fed on a semi-synthetic diet (Akronom Ltd., Budapest, Hungary) and tap water ad libitum [32]. The animals received humane care according to the criteria outlined in the UK “Guide for the Care and Use of Laboratory Animals”, authorized by the Ethical Committee for Animal Research, University of Debrecen [33]. Five randomly chosen animals were present in each group.

### 2.5. Aerogel Treatment and Autopsy

The FSGM suspended in physiological saline were injected precisely and carefully into the upper abdominal cavity of the animals. Standard 23G × 1 1/4” needles were used with 1 mL syringes. Two different concentrations were applied: 52 or 104 mg FSGM in dry weight per kilogram body weight. The two different groups of treated mice are termed “low dose” and “high dose”. All mice were treated with FSGM 2 times a week for 3 weeks, totaling 6 times. Additionally, the control group received an equivalent volume of saline administered in the same way.

These experiments aimed to investigate the short-term biodistribution of FSGM in order to determine their applicability as vehicles for antimetastatic drugs. Furthermore, the parathymical metastasis mouse model used in the related study required a 3 weeks interval of treatment [14].

The weights of mice were measured before each injection of FSGM. At the end of the experiment, the mice were sacrificed with cervical dislocation and an autopsy was performed. During the autopsy, the abdominal cavity for each mouse was opened, peritoneal organs (liver, spleen), the thymus and other tumor-associated organs (intestine, colon) were removed and fixed in 4% buffered formaldehyde. The specimens were embedded in paraffin, sectioned, stained with hematoxylin–eosin (H&E) and hematoxylin monochrome dye. The prepared specimens were examined under brightfield and fluorescence microscopes. Histological studies of abdominal organs and lymphatic tissues were performed at the Department of Pathology, Kenézy University Hospital of Debrecen.

### 2.6. Fluorescence Microscopy

The biodistribution of FSGM in tissue samples was investigated with fluorescence microscopy. Parallel to the conventional H&E staining, we applied hematoxylin monochrome staining on the tissue samples to avoid the autofluorescence of eosin dye. The tissue samples were examined with 3 different fluorescence filter sets—DAPI (EX: 375 nm, EM: 460 nm), FITC (EX: 480 nm, EM: 535 nm) and Texas Red (EX: 560 nm, EM: 630 nm)—in order to determine the localization of FSGM in the tissues. The green fluorescence of FSGM at an excitation wavelength of 480 nm was applied to track the microparticles in the tissue samples with a Motic AE31 fluorescence microscope.

### 2.7. Immunohistochemistry

The immunohistochemical staining of CD68 and CD163 (BioCare Medical, Pacheco, CA, USA) was used on formalin-fixed paraffin-embedded tissues to detect monocytes/macrophages. Serial sections of 5 µm thick tissue slices were cut from paraffin blocks. Heat-induced antigen retrieval was performed with Reveal Decloaker (CD68) and BioCare’s Diva Decloaker (Biocare Medical, Pacheco, CA, USA). Endogenous peroxidase activity was blocked with BLOXALL blocking solution (SP-6000). For the detection of mouse primary antibodies on murine tissue M.O.M.^®^ (Mouse on Mouse) immunodetection kit was applied. An enzyme-conjugated avidin/streptavidin (VECTASTAIN^®^ ABC) complex was used in the chromogen detection of the staining procedure, while cell nuclei were counterstained with hematoxylin–eosin. Tissue sections were finally mounted in a permanent mounting medium (Histolab, Göteborg, Sweden).

## 3. Results and Discussion

### 3.1. Biocompatibility of Aerogel Microparticles

All mice were healthy following the FSGM suspension treatments during the 3-week experiment time. No reduction was observed in food intake and no physiological defects or dysfunctions were detected. There was no significant change in the weight of the animals within any group at the end of the treatments (Figure S2).

One of the most important findings of the autopsy is that the hybrid aerogel microparticles (FSGM) were not present at the site of injection or anywhere visible in the abdominal cavity at the end of the experiment. The only exception was a random case when a minor fraction of the particles formed a deposit on the outer surface of the intestine (Figure S3). This deposit was detected on the mesenterial surface in one animal, but no particles were detected inside the mesenterial connective tissue in any animal.

**Figure 3:**
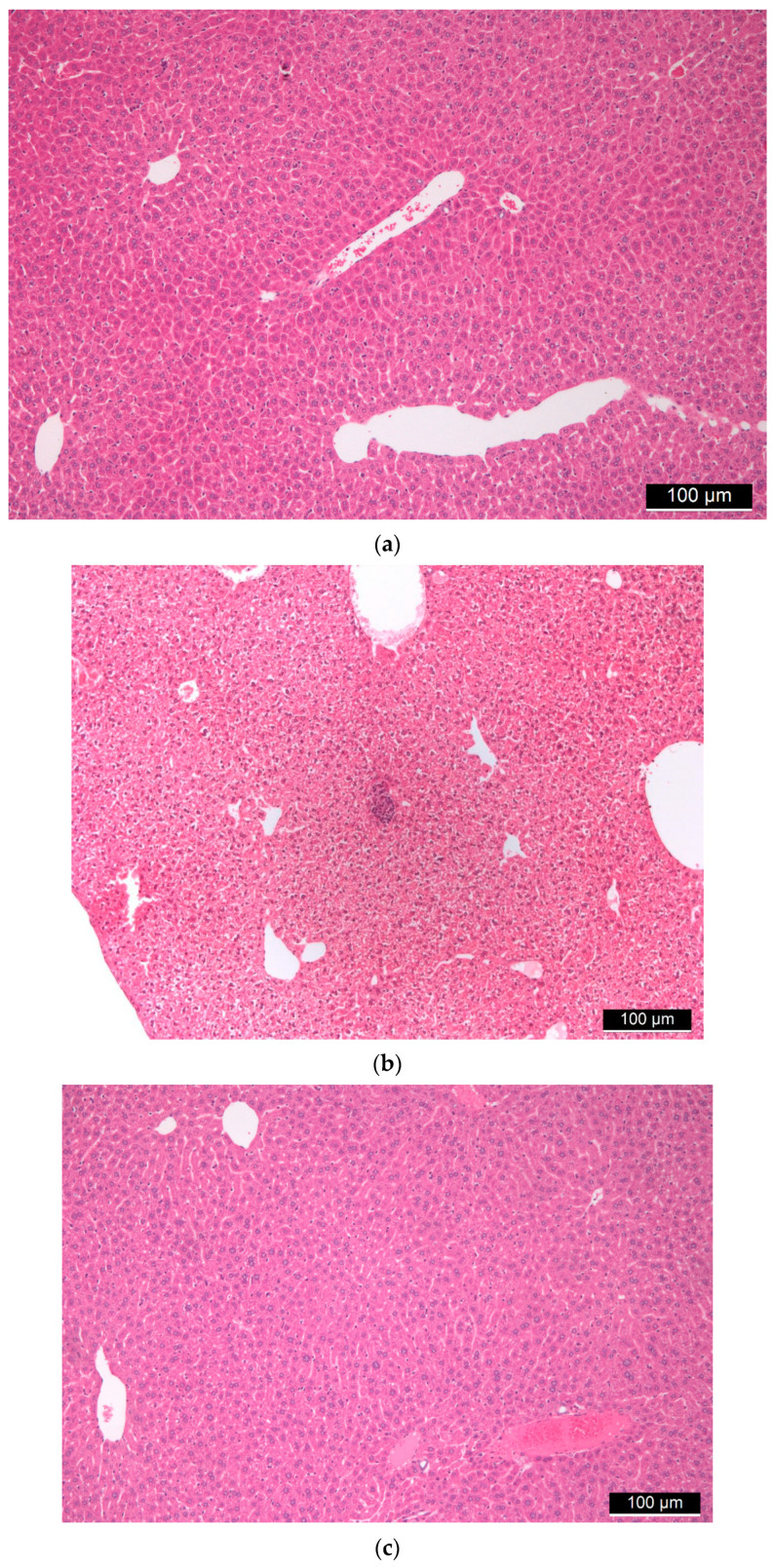
(**a**) Control liver tissue. (**b**,**c**) Liver tissues of “low dose” (**b**) and “high dose” (**c**) FSGM-treated mice.

Liver tissues from all mice underwent histological examinations, during which hematoxylin–eosin staining was performed. In these tissues, the basic structure of the liver was preserved, furthermore both the central veins and the portal fields were recognizable. In the case of higher doses of FSGM, signs of stagnation were seen in some places centrally and sinusoidally. The artery, vein, and bile duct were retained in the portal fields. There were no clear tissue indications in the direction of chronic inflammation or granulomatous inflammation. No foreign body type granuloma or other granulomas were observed. Hyperplasia, cholestasis, or inflammatory cells did not occur on the bile ducts. Neither endothelial damage nor microthrombi was found in the vessels. Hepatocytes showed the usual trabecular arrangement, there was no significant change in their size. Glycogen was uniformly observed everywhere in the cytoplasm both around the central veins and the portal fields. Signs of uni- and multivacoular parenchymal degeneration, furthermore any unicellular, group or diffuse necrosis was not visible. The size and staining of the nuclei were uniform; the nuclei membranes of the cells were uniformly thin. Additionally, the chromatin structure was uniformly finely granular and the nucleolus of the cells was not conspicuous. There were no binuclear regenerating hepatocytes. Kupffer cells and hematopoietic cells were rarely visible sinusoidally. No signs of allergic inflammation could be observed (Figure 3).

**Figure 4:**
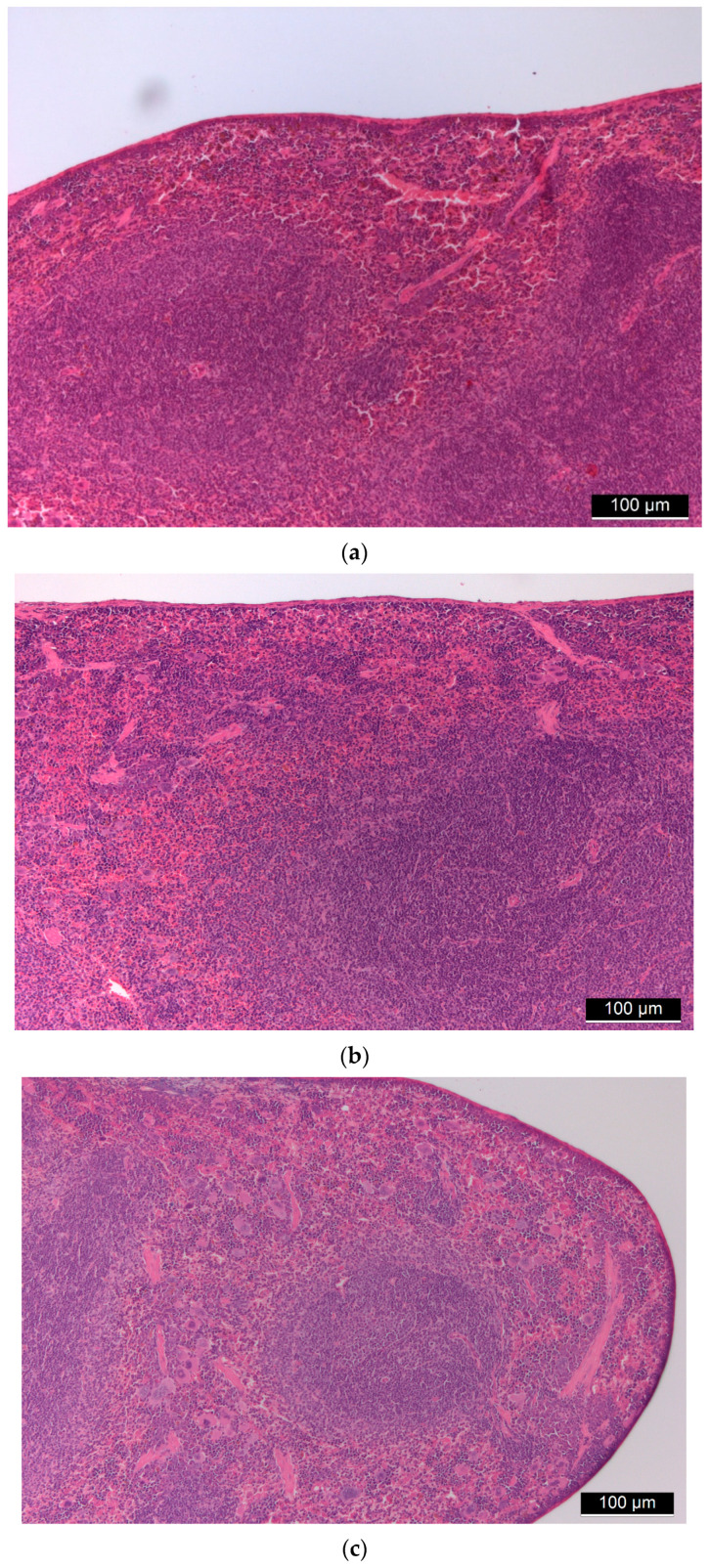
(**a**) Control spleen tissue. (**b**,**c**): Spleen tissues of “low dose” (**b**) and “high dose” (**c**) FSGM-treated mice.

The basic structure of the spleen was intact in all animals, the white pulp with lymphatic follicles was observable as well as the sinusoidal system of the red pulp was visible with degrading red blood cells. There were no signs of granulomatous inflammation and septic condition (Figure 4).

**Figure 5:**
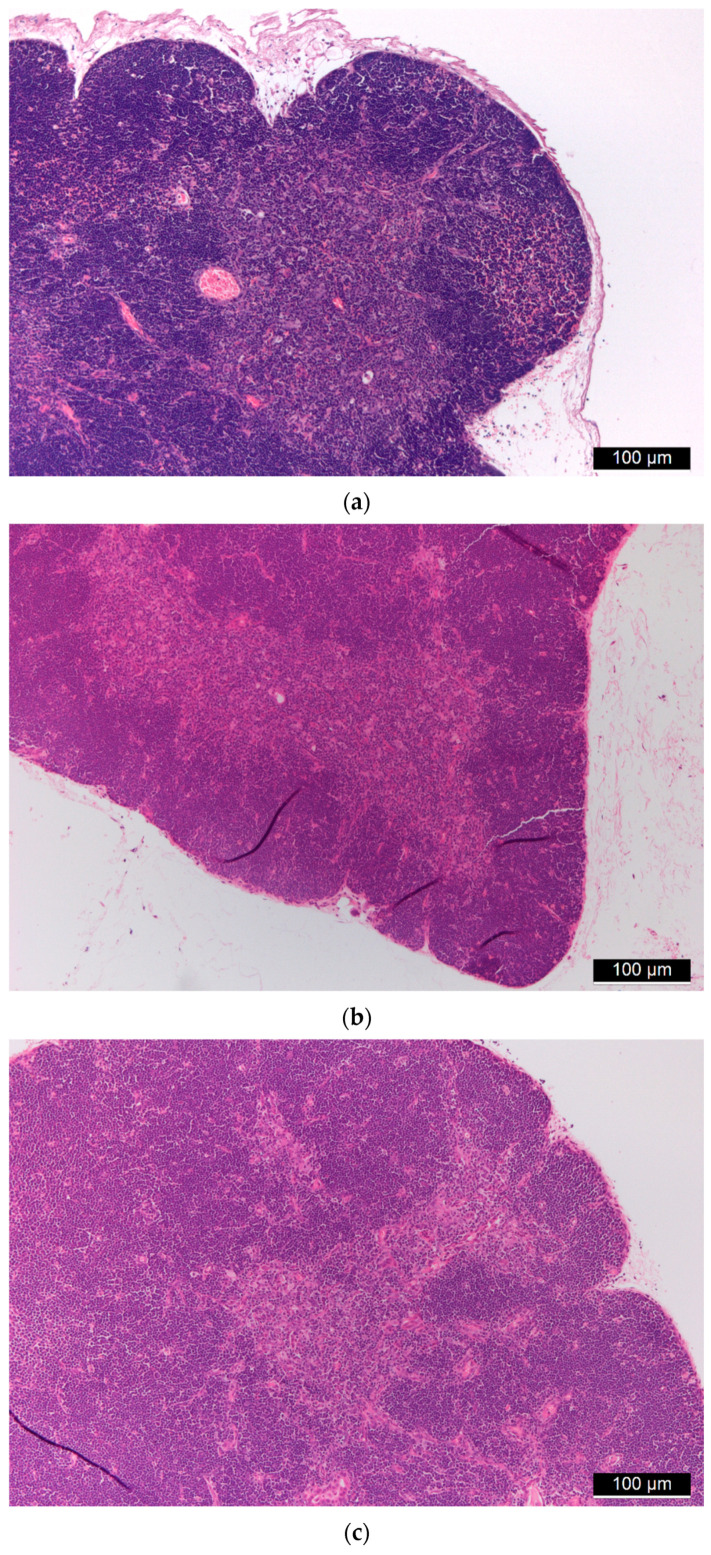
(**a**) Control thymus tissue. (**b**,**c**): Thymus tissues of “low dose” (**b**) and “high dose” (**c**) FSGM-treated mice.

The thymus tissues of both control (Figure 5a) and treated (Figure 5b,c) animals consisted of a brighter medulla without any alterations and a darker, non-abnormal cortex with immature lymphoid elements. At low magnification, their usual lobular structure was also retained. A granulomatous process, which could indicate the presence of foreign material, or histiocyte proliferation was not visible. The basic structure of the mediastinal (parathymic) lymph nodes observed in many animals was preserved. Granulomatous lymphadenitis or other lymphadenitis was not visible. Immunohistochemical markers CD68 and CD163 were used for the detection of histiocytes belonging to normal cells, as detailed later.

Other organs were also investigated, but no FSGM were observed outside the peritoneum. Only the mediastinal lymphatic pathway was affected, as detailed in the next section.

### 3.2. Biodistribution of Aerogel Microparticles

In our previous work, black ink particles were injected into the peritoneum of C3H mice. After 24 h the mice were sacrificed and an autopsy was performed. The thymus of the mice was removed and standard procedures applying H&E staining were used for the histological evaluation. The results showed that ink particles traversed the diaphragm and accumulated in the cortical part of the parathymic lymph nodes. It was also shown that metastatic cancer cells followed the same route, most probably via lymphatic circulation [14].

**Figure 6:**
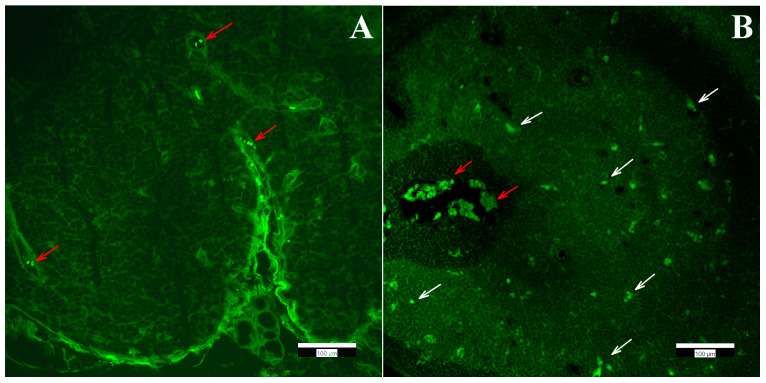
Panel (**A**): Autofluorescence of red blood cells (red arrows) inside control thymus tissue. Panel (**B**): Fluorescence of FSGM (white arrows) in “high dose”-treated thymus tissue. Scale bars: 100 µm.

In order to obtain qualitative information on the distribution of FSGM, we used fluorescence microscopy and immunohistochemical staining. The presence of FSGM was investigated in different organs (liver, kidney, spleen, thymus) with a fluorescence microscope. The observation of the tissue samples with the FITC fluorescence filter clearly showed the presence of the FSGM in the lymph node of the mouse thymus, and the absence of the FSGM in the mouse liver, kidney and spleen. In the case of mouse lymphatic tissue, next to the signal of the FSGM, the natural autofluorescence of the tissue was observed. The autofluorescence of the lymphatic tissues has been characterized in numerous previous studies [34,35]. Granular cells have a well-defined autofluorescence signal when applying the standard filter sets in the fluorescence microscope. In order to discriminate the signal of the FSGM from the background autofluorescence, an image processing method was implemented. Images were taken from the same sample applying different filter sets, and the images taken with the DAPI and Texas Red filters were subtracted from the FITC filtered images. The resulting image clearly shows the localization of the FSGM inside the tissue (Figure 6). The application of this image processing method for every other tissue sample verified that neither the liver, nor the spleen, nor the kidney contained FSGM, only the thymus.

The observations in combination with the findings of previous studies carried out with ink dye particles suggest that the disappearance of the silica–gelatin aerogel microparticles from the abdominal cavity of mice is most probably due to their entrance to the lymphatic circulation. If the FSGM enter the lymphatic circulation, it is logical to assume that a large number of the microparticles will eventually localize in the parathymic lymph nodes, because they fulfill the role of sentinel nodes in the current context.

### 3.3. Immunohistochemical Staining

**Figure 7:**
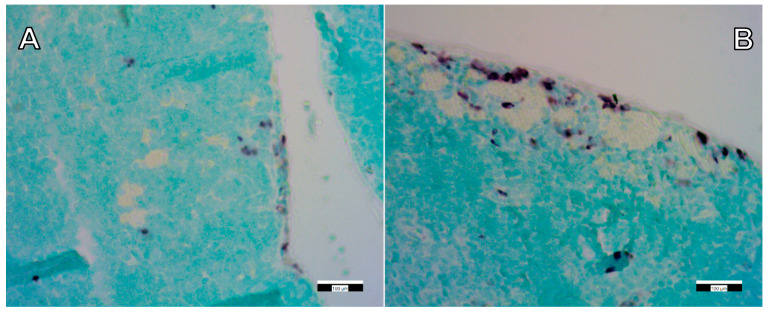
Immunohistochemical staining (CD68) of control (**A**) and “high dose” FSGM-treated (**B**) thymus tissue. Scale bars: 100 µm.

**Figure 8:**
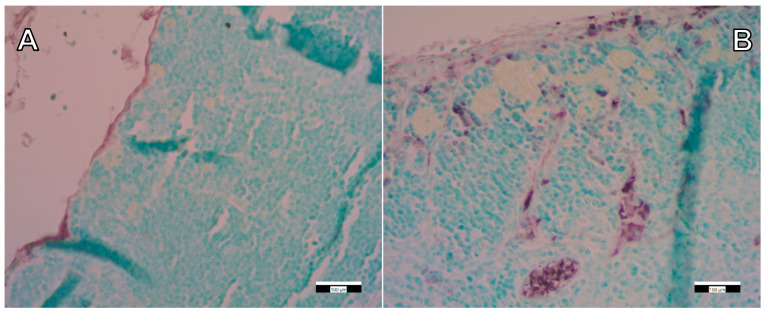
Immunohistochemical staining (CD163) of control (**A**) and “high dose” FSGM-treated (**B**) thymus tissue. Scale bars: 100 µm.

Following the detection and identification of the FSGM in the thymus tissue, immunohistochemical staining was performed to detect immune cells (macrophages) surrounding the FSGM. This proved that the presence of silica-containing FSGM activates the circulating immune cells in the thymus (Figure 7 and Figure 8). Still, it is important to emphasize that the applied concentrations of FSGM did not cause detectable toxicity or inflammation in any of the investigated organs.

### 3.4. Toxicity of Mesoporous Silica Nanoparticles and Aerogels as Comparison

Mesoporous silica nanoparticles have intensively been studied in drug delivery. Viability studies have proven that at low concentrations, these particles are biocompatible [36,37,38]. For example, Lu et al. showed that fluorescently-labeled, Camptothecin-containing silica nanoparticles accumulated in tumors and had tumor-suppressing effects in xenograft models [37]. However, the intraperitoneal and intravenous injections of nanoparticles showed systemic toxicity in some cases [38]. Furthermore, mesoporous silica nanoparticles are not biodegradable [39]. Accumulation of silica in tissues may cause the accumulation of immune cells causing inflammation, notably silicosis [40]. The physicochemical properties of the nanoparticles, including their particle size distribution, shape, surface area and morphology, have been proven to play important roles in their biocompatibility and biodistribution, together with the charge and surface chemistry of the particles [36,41]. Yang et al. reported the correlation among size, surface modification and administration route of the applied nanoparticles with the toxicology of the particles [42]. The composition of nanomaterials can be modified to reduce their toxicity and undesirable side effects, e.g., by hybridization with a biopolymer resulting in biodegradable, biocompatible and therapeutically attractive biomaterial [43].

We propose that the good biocompatibility of the silica–gelatin aerogel microparticles reported in this study is the combined consequence of their high gelatin content and the significant increase in the size of the porous material, i.e., the formulation of microparticles instead of nanoparticles.

There is only a limited number of in vivo toxicity studies available on aerogels that could serve for comparative evaluation of the results presented in this paper. In a relevant pilot study, the in vivo biocompatibility of millimeter-sized polyurea crosslinked silica aerogels were studied as subcutaneous and intramuscular implants in Sprague–Dawley rats. The longest incubation time was 20 months. During this time, the aerogels were well tolerated, and no toxicity was detected in the tissues surrounding the aerogels. The histological evaluation of distant organs showed no toxicity [24].

## 4. Conclusions

The biodistribution pathways and the physiological effects of fluorescein-labeled silica–gelatin aerogel microparticles (FSGM) were investigated by injecting their suspension into the peritoneum (abdominal cavity) of healthy C3H mice. In general, the injected FSGM were well tolerated over the 3-week-long acute toxicity experiments. Neither the 52 mg kg^−1^ nor the 104 mg kg^−1^ concentration caused toxicity in the animals. No reduction was observed in food intake, no physiological defects or dysfunctions were detected, and all mice were healthy at the end of the experiments. The autopsy revealed the important finding that the FSGM were not present at the site of injection in the abdominal cavity of the animals at the end of the experiment. The histological study of abdominal organs (liver, spleen, kidneys, thymus) and lymphatic tissues showed no signs of toxicity. The localization of the FSGM in the organs was studied by fluorescence microscopy. Aerogel microparticles were not detected in any of the abdominal organs, but they were clearly visible in the cortical part of the parathymic lymph nodes, where they accumulated. The accumulation of the aerogel microparticles in parathymic lymph in combination with their absence in the reticuloendothelial system organs, such as the liver or spleen, suggests that FSGM entered lymphatic circulation. A similar biodistribution pathway was observed for dry ink particles injected into the abdominal cavity of mice. The entrance of silica–gelatin aerogel microparticles into the lymphatic circulation can be exploited to design passive targeting drug delivery systems for flooding metastatic pathways of abdominal cancers that spread via the lymphatic circulation.

## Data Availability

The complete sets of data presented in this study are available on request from the corresponding author.

## Supplementary Materials

The following are available online at https://www.mdpi.com/article/10.3390/ijms22189756/s1.
