# Supplementary material for: Mesoporous Aerogel Microparticles Injected into the Abdominal Cavity of Mice Accumulate in Parathymic Lymph Nodes
Source: Int J Mol Sci. 2021 Sep 9;22(18):9756. doi: 10.3390/ijms22189756 (PMC8465913; doi:10.3390/ijms22189756)
Supplement: Supplementary file 1 [file ijms-22-09756-s001.zip › ijms-1371725-supplementary.pdf]

Supporting Information

for

Mesoporous Aerogel Microparticles Injected into the Abdominal Cavity of Mice Accumulate in Parathymic Lymph Nodes

Gábor Király[a], John Chinonso Egu[b], Zoltán Hargitai[c], Ilona Kovács[c], István Fábián[b], József Kalmár[b,*], Gábor Nagy[a]

[a] Department of Molecular Biotechnology and Microbiology, University of Debrecen, Egyetem tér 1, Debrecen, H-4032, Hungary

[b] Department of Inorganic and Analytical Chemistry, MTA-DE Homogeneous Catalysis and Reaction Mechanisms Research Group, University of Debrecen, Egyetem tér 1, Debrecen, H-4032 Hungary

[c] Department of Pathology, Kenézy University Hospital, University of Debrecen, 2-28 Bartók Béla Street, Debrecen, H-4031, Hungary

* Corresponding author: e-mail: kalmar.jozsef@science.unideb.hu

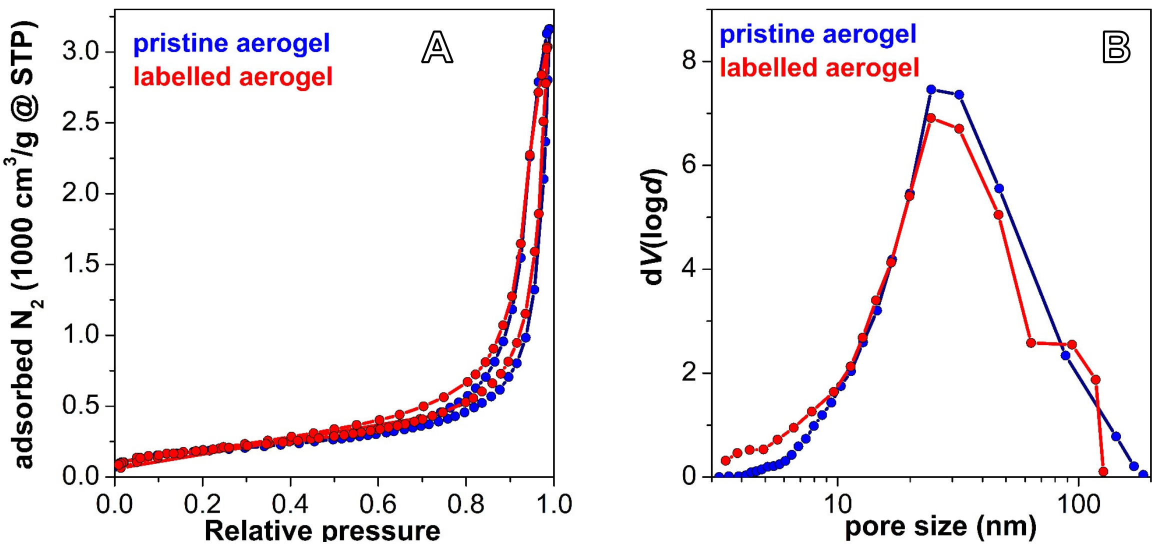

Figure S1. Nitrogen adsorption-desorption isotherms (A) and pore size distributions (B) calculated from the desorption curves by the BJH method.

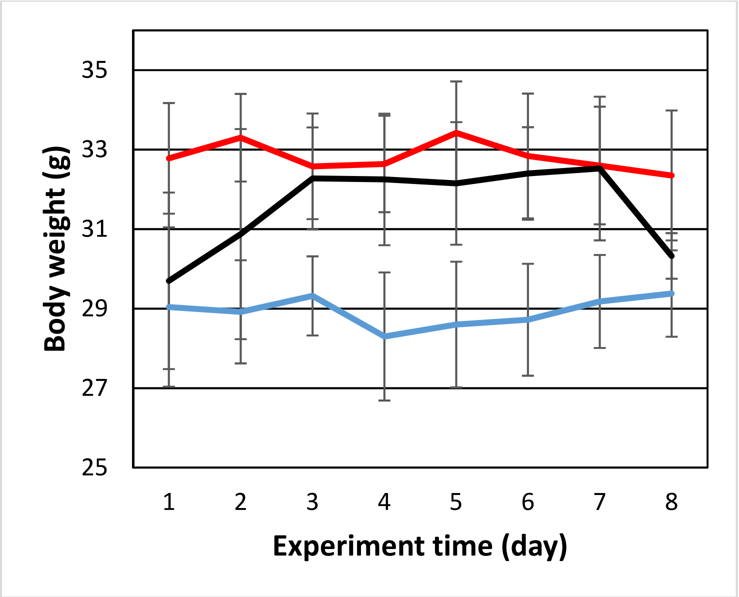

Figure S2. Average body weights of control (blue) and FSGM treated mice (red: "high dose", black: "low dose"). The results are presented as mean values ± the standard deviation (SD). The starting weight of the control animals was less than the weight of the treated mice due to their younger age.

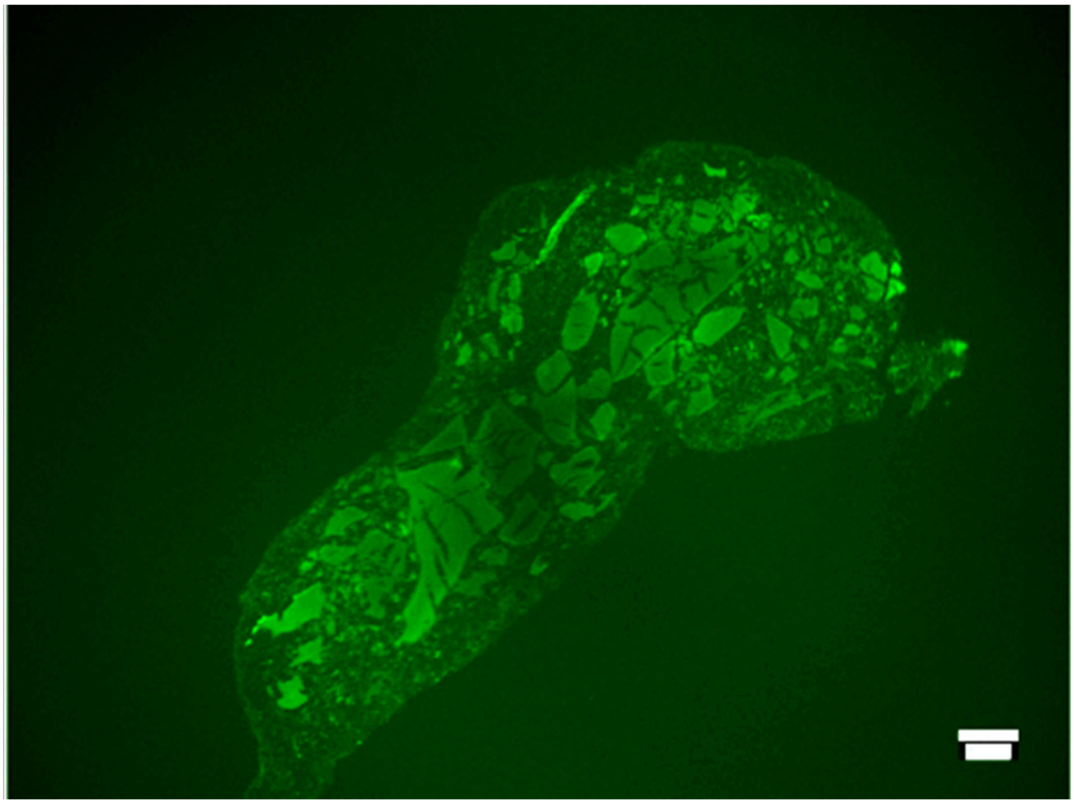

Figure S3. FSGM particle aggregation recovered from next to the intestine of C3H mouse. Fluorescent images were taken with FITC filter. Scale bar: 100 µm.
